# Investigating the potential effects of olive leaves and ginger rhizome extracts on kidney and liver functions in alloxan-induced diabetic rats

**DOI:** 10.22038/ajp.2024.24986

**Published:** 2025

**Authors:** Shatha Alshaer, Mohammad A. A. Al-Najjar, Feras Darwish El-Hajji, Reem Abu-Tayeh

**Affiliations:** 1 *Pharmaceutical Sciences and Pharmaceutics Department, Applied Science Private University, Amman-Jordan*; 2 *Clinical Pharmacy and Therapeutic Department, Applied Science Private University, * *Amman-Jordan *; 3 *Pharmaceutical Chemistry and Pharmacognosy Department, Applied Science Private University, Amman-Jordan*

**Keywords:** Diabetes mellitus, Alloxan, Kidney function, Liver function, Olive leaves, Ginger rhizome

## Abstract

**Objective:**

Although there are many drugs on the shelves of pharmacies to manage diabetes mellitus (DM), many people around the world still use herbal preparations to treat it. This study investigated the effect of an aqueous combination of olive leaves and ginger rhizome extracts on type 1 diabetes mellitus (T1DM) using various physiological markers.

**Materials and Methods:**

Fifty-two Wistar rats were distributed into 2 healthy and 6 diabetic groups. Forty rats were given alloxan (150 mg/kg) as an intraperitoneal single-dose to induce T1DM. Treatments including insulin with/without individual and combined extracts, were started 4-day post-induction. The extracts were administered orally (500 mg/kg) and insulin was administered subcutaneously (6 IU/kg) in single-doses once a day. After one week of treatment, the blood samples were collected to measure Fasting blood glucose (FBG), Alanine aminotransferase (ALT), Alkaline phosphatase (ALP), and creatinine.

**Results:**

The diabetic group that received the combination of both extracts with insulin had a lower mortality rate after 14 days of treatment. The diabetic group receiving insulin with the olive leaves extract, demonstrated a decrease in ALT levels to 33.7 U/L (p=0.345) while maintaining the ALP levels within the normal range 126.9 U/L (p=0.463). Creatinine was significantly reduced to 1.1 mg/dl (p=0.028) and 0.7 mg/dl (p=0.028) in diabetic groups that received individual olive leaves and ginger extracts with insulin respectively.

**Conclusion:**

To conclude, this combination with insulin had powerful effects to improve the mortality rate in diabetic rats over other groups, and the two extracts separately were able to decrease the creatinine levels.

## Introduction

Diabetes mellitus (DM) is a severe, long-term disorder, characterized by a rise in blood glucose levels. Currently, DM is one of the fastest universal health disasters growing within the current century and is one of the top ten major causes of death globally in adults (Abouzid et al., 2022; Madmoli et al., 2019; Saeedi et al., 2019; Zheng et al., 2017) 

There are several types of DM such as type 1 diabetes mellitus (T1DM) and type 2 diabetes mellitus (T2DM) (Zarei et al., 2015). T1DM is a common chronic disease in children, and it has been rapidly growing globally ((Yeung et al., 2011; Abouzid et al., 2022). According to 2019 statistics, there are 1.1 million children and adolescents (aged under 20 years) affected by T1DM worldwide (Atlas, 2019).

T1DM previously termed insulin-dependent diabetes (IDD), occurs due to impaired β-cells of the pancreas (Kazemian et al., 2015). It originates from complex etiologies of genetic disposition, the immune system, and environmental influences (Yeung et al., 2011). Noticeably, diet over the last few decades has been hypothesized as a major driver of T1DM (Needell and Zipris, 2016). Furthermore, obesity has been associated with T1DM patients, in comparison to their peers without T1DM (Minges et al., 2013). Medicinal agents such as injectable insulin and oral hypoglycemic drugs exist in the market to control and manage DM. However, to the present, no complete recovery from the disease has been achieved (Dwivedi and Daspaul, 2013). Despite the drugs’ efficacy, the challenges remain including side effects, cost, and availability especially in middle, and low-income countries (Yatoo et al., 2017). Insulin therapy is administered as multiple daily injections or as a continuous subcutaneous infusion in DM patients (Balducci et al., 2014). Whilst evidence supports insulin efficacy, patients are concerned about weight gain, the threat of hypoglycemia, fear, and pain associated with injections (Kim et al., 2017). To overcome the challenges of either the unavailability or cost of the drugs, several plants with hypoglycemic effects have been tested and /or used by diabetic patients worldwide (Ibrahim and Al-Shathly, 2015). Many *in-vivo* studies conducted to investigate the antidiabetic effect of plants showed a powerful effect on managing DM, especially plants that have phenolic compounds (Bindu and Narendhirakannan, 2019; Eddouks et al., 2017). Furthermore, clinical studies resulted in successfully commercializing many of the plant extracts or their active constituents in the form of supplementary drugs like CinnaBetic II (cinnamon water extract capsules) (David et al., 2015). However, the use of natural products is still limited due to the lack of rigorous studies that investigate the potential synergistic effects of combining more than one beneficial herbal plant. 

The olive tree (*Olea europaea*) is a perennial green tree that belongs to the family Oleaceae and grows in warm-temperature areas of the world (Ghanbari et al., 2012). Olive leaves have many bioactive phenolic compounds that have been studied for their antioxidant, anti-hypertensive, and hypoglycemic potential activities (Ghanbari et al., 2012; Liu et al., 2014). Recent studies propose that the mechanism of the antidiabetic effect of the olive leaves extract could be via inhibition of pancreatic amylase activity, deterrence of starch breakdown, inhibition of glucose uptake, and /or stimulation of hepatic glycogen synthesis (Bi et al., 2017; Guex et al., 2019).

*Zingiber officinale* *, *known as ginger, belongs to the family Zingiberaceae and is a valuable hot spice perennial plant indigenous to South Asia. The rhizomes of ginger have been traditionally used for the treatment of hypertension, DM, sore throats, and infectious diseases (Bindu and Narendhirakannan, 2019). Recent studies showed that the polyphenolic compounds in ginger have glycemic regulatory activities such as inhibiting α-amylase and α-glucosidase enzymes in carbohydrate digestion and increasing insulin release and sensitivity (Bi et al., 2017; Yang et al., 2024)

A combination of synthetic drugs complemented with herbal extracts could be a new and highly effective therapeutic approach to control hyperglycemia (Prabhakar et al., 2014) if it plays one or more types of synergism effects such as:

 1) Increasing the efficacy of the therapeutic agent,

(2) Lowering the dose is needed to achieve a controlled sugar level. Decreased dosage could lead to decreased side effects, and (3) Minimizing or slowing down the development of drug resistance (Prabhakar et al., 2014).

Until now there are no studies that investigated the antihyperglycemic effect of olive leaves and ginger rhizome in a combined formulation. Thus, this study is the first study that was done to investigate the effect of a combination consisting of aqueous olive leaves extract and aqueous ginger rhizome extract on T1DM using different physiological markers fasting blood glucose (FBG), alanine transaminase (ALT), alkaline phosphatase (ALP), and creatinine. 

## Materials and Methods

This study was ethically approved by the Applied Science Private University Ethics Committee for the Care and Use of Experimental Animals in Education and Scientific Research – Faculty of Pharmacy (Approval No. 2020-PHA-10).

### Plants and their extractions

Olive leaves were picked in October 2019, from an olive tree plantation farm in Jerash, Jordan. Fresh ginger rhizomes were bought from the local market in Amman, Jordan.

Aqueous extracts of the plants were freshly prepared manually in the laboratory and used as test substances for treatment groups. The extraction method of olive leaves was adopted by Zoair, and Abu-zaiton and Abu-Albasal with slight modifications. To prepare the aqueous olive leaves extraction, 10 g of the olive leaves was weighed, cut in an electric blender (Model No. MX – GX1521), and then soaked in 100 ml distilled water in a beaker for 24 hr. The whole mixture was boiled for 3 min and then filtered to get the final extract with a concentration of 100 mg/ml (Abdulrazaq et al., 2012; Zoair, 2014; Abu-zaiton and Abu-Albasal, 2012a).

For the ginger rhizomes, the method of extraction was adopted from Abdulrazaq with slight modifications. Briefly, 10 g of fresh ginger rhizomes was crushed in an electric blender, then soaked in 100 ml distilled water for 24 hr at room temperature and filtered to get 100 mg/ml of the ginger extract (Abdulrazaq et al., 2012). Finally, the two extracts were kept individually in closed amber glass bottles in the fridge (at 2 -8ºC), until used.

### Experimental animals and experimental design

Fifty-two, eight-week-old, healthy Wistar rats of both sexes with a weight range of 150 to 225 g were housed at a temperature of 21– 23^o^C and humidity of 35- 70 %, in controlled rooms, with 12 hr light – 12 hr dark cycles. Rats were placed in standard clear-sided cages according to their gender to avoid gestation. Wood shavings were used as bedding and rats were fed a commercially available diet (Local Supplier, Jordan) and freshwater was freely offered. The adaptation period for the rats was two weeks before the start of the experiment.

### Induction of diabetes

The rats were fasted overnight and blood glucose levels were measured before inducing DM by using ACCU-CHEK glucometer (Performa, ROCHE, Penzberg, Germany) (Kumbhare and Sivakumar, 2019). A single dose of 150 mg/kg alloxan monohydrate (purchased from Santa Cruz Biotechnology., California, USA) was administered to each rat via intraperitoneal injection (IP) to induce T1DM. The alloxan dose was dissolved in a sterile saline solution (0.9% sodium chloride, pH 7) directly before use (Aluwong et al., 2016; Hassanpour Fard et al., 2015). Each rat received 1-2 ml of 5% glucose solution orally directly after alloxan injection. The rats had free access to food 30 min after the alloxan injection and the bottles of glucose solution were kept in the cages for 24 hr to prevent fatal hypoglycemia (Abu-zaiton and Abu-Albasal, 2012a). Four days after alloxan injection, the overnight FBG was recorded by using an ACCU-CHEK glucometer (Kumbhare and Sivakumar, 2019). 

Extracts given as treatment substances (in groups IV to VIII) were administered orally once a day and insulin was given 6 IU/kg subcutaneously as a single dose (in groups III to VI). The Novo Nordisk pen was used for the injection of the appropriate dose of Mixtard 30 insulin (Novo Nordisk, Bagsvaerd, Denmark) (Table 1).

### Samples collection and preparation for further analyses

#### Urine samples

The urine samples were collected from all rats within target groups 4 days after alloxan injection before any treatment by slightly pressing the tail and back of the rat to collect fresh urine into a polystyrene beaker (Kurien et al., 2004; Prasad et al., 2009)

### Blood samples

Blood samples were withdrawn from all groups (treatment and control) at three points of time during the study: the first withdrawal was on day zero before inducing the DM animal models, the second blood sample withdrawal was on Day 4 post-alloxan injection, and the third blood sample was drawn after one week after treatment initiation from all surviving rats (Day 11).

All blood samples were collected at 10 a.m. from retro-orbital sinus veins of overnight fasting Wistar rats (after 18 hr of a fasting state) (Khalil et al., 2014).

For ALT, ALP, and creatinine serum levels analyses, blood samples were centrifuged at 2000 g for 15 min using microcentrifuge (Biosan, USA), and serum was separated and stored at -40ºC into a deep freeze for further testing of biochemical parameters (Burns and de Lannoy, 1966; Khalil et al., 2014; Kumbhare and Sivakumar, 2019). 

### Biological evaluations of treated rats

#### Evaluation of ketone body level

To assess the semi-quantitative levels of ketone bodies in urine by visual record, the Combur strips test was used; it was purchased from (ROCHE, Penzberg, Germany) (Prasad et al., 2009).

#### Evaluation of fasting blood glucose level

A drop of the blood sample of each rat was introduced directly into the glucose strip to measure the FBG level using the ACCU-CHEK glucometer, then the FBG level was recorded.

#### Evaluation of liver function

Ultraviolet enzymatic kits for ALT (Linear (Barcelona, Spain)) and ALP (Biosystems (Barcelona, Spain)) were used to detect serum levels. The analysis was done following the instruction protocol. 

#### Evaluation of kidney function

Ultraviolet enzymatic assessment of serum creatinine levels was done using a bioanalytical kit (Spinreact, Barcelona, Spain), and the procedures were precisely followed as written in the instruction protocol.

### Statistical analyses

All data were collected in a Microsoft Excel Office 365 spreadsheet and then analyzed using SPSS^©^. Data is presented as mean ± S.D (Standard Deviation).

Non-parametric tests were adopted using the Wilcoxon Signed Ranks Test and Kruskal-Wallis statistical test, with p-values ≤0.05 considered significant differences within a group or between groups that received treatment. One-Way ANOVA: *Post Hoc* Multiple Comparisons (Tukey equal variance assumption) analysis was applied to test for differences among groups.

Survival of animals was compared after 1 week (Day 11) and 2 weeks (Day 18) of different treatments, using Pearson Chi-square test, p≤0.05 indicating a significant difference among the groups regarding the survival of animals.

## Results

### Alloxan-induced diabetes and ketone bodies test

On day 4 post-alloxan administration, FBG levels were elevated at least three times in all groups that received it compared with baseline FBG for each group (Table 2). This increase in FBG levels indicates the development of DM. 

Notably, alloxan was able to induce the production of ketone bodies in rats' urine, as proved by the change of color of the Combur strips test. The elevation of ketone body concentration in urine indicates the development of T1DM.

### Survival of the animals

**Figure 1:**
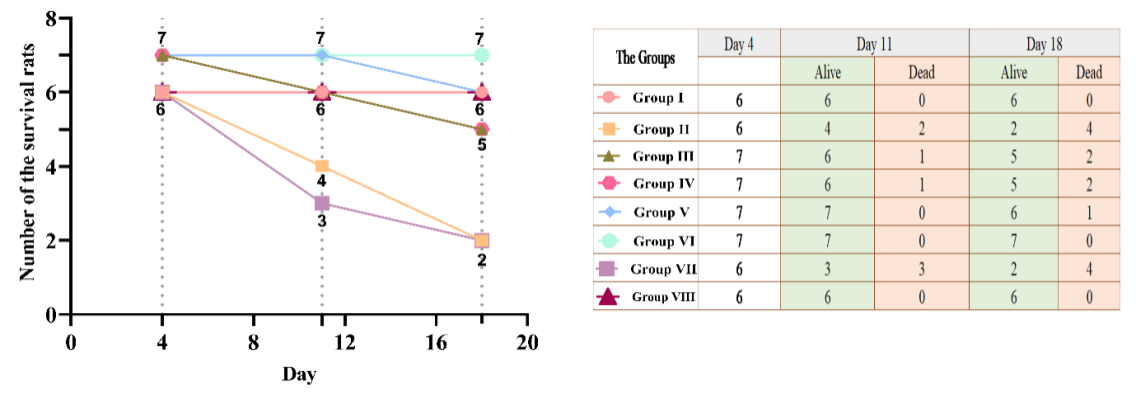
Comparative survival of the rats, Day Zero (before alloxan treatment, initial), 4 days after alloxan treatment (Day 4), and 1 week after different treatments (Day 11). Group I (Healthy, without treatment, n=6), Group II (Diabetic, without treatment, n=6), Group III (Diabetic, 6 IU/kg Insulin, n=7), Group IV (Diabetic, 6 IU/kg Insulin + 500 mg/kg Olive leaves extract, n=7), Group V (Diabetic,6 IU/kg Insulin + 500 mg/kg Ginger rhizome extract, n=7), Group VI (Diabetic,6 IU/kg Insulin + 500 mg/kg Olive leaves extract + 500 mg/kg Ginger rhizome extract, n=7), Group VII (Diabetic, 500 mg/kg Olive leaves extract + 500 mg/kg Ginger rhizome extract, n=6), and Group VIII (Healthy, 500 mg/kg Olive leaves extract + 500 mg/kg Ginger rhizome extract, n=6).

The number of surviving animals was followed up for the whole period of the study as shown in (Figure 1). The mortality rate showed no significant difference among the different groups in the first week (p=0.235). However, by the end of the second week, the mortality rate among different groups showed a statistically significant difference (p=0.005). Group I and Group VI had a notably better survival rate than other groups. Because the mortality rate was higher in the third week, comparisons were of no value. Accordingly, it was decided to conduct the rest of the comparative tests on the first week (Day 11) results only, to produce statistically reasonable comparisons.

### Fasting blood glucose analysis

All groups that received insulin (groups III to VI) had a significantly decreased FBG on day 11 compared to day 4 (p=0.007). The groups with decreased FBG more than others were determined by One-Way ANOVA: Post Hoc Multiple Comparisons (Tukey equal variance assumption). FBG decreased in all groups that received insulin (with or without extracts of olive leaves and ginger) more than in other groups, however, they had no statistically significant differences between each other. Additionally, group VIII exhibited a significantly decreased level of the FBG after one week of treatment, the difference was statistically significant compared to the healthy group without treatment (Table 2).

### Liver functions analyses

#### Serum Alanine Aminotransferase (ALT) analysis

**Figure 2:**
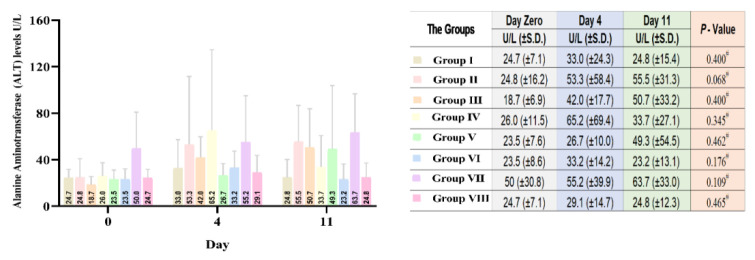
Comparative average ALT serum levels in the studied groups, Day Zero (before alloxan treatment, initial), 4 days after alloxan treatment (Day 4), and 1 week after different treatments (Day 11). Group I (Healthy, without treatment, n=6), Group II (Diabetic, without treatment, n=6), Group III (Diabetic, 6 IU/kg Insulin, n=7), Group IV (Diabetic, 6 IU/kg Insulin + 500 mg/kg Olive leaves extract, n=7), Group V (Diabetic,6 IU/kg Insulin + 500 mg/kg Ginger rhizome extract, n=7), Group VI (Diabetic,6 IU/kg Insulin + 500 mg/kg Olive leaves extract + 500 mg/kg Ginger rhizome extract, n=7), Group VII (Diabetic, 500 mg/kg Olive leaves extract + 500 mg/kg Ginger rhizome extract, n=6), and Group VIII (Healthy, 500 mg/kg Olive leaves extract + 500 mg/kg Ginger rhizome extract, n=6).

The mean reading of serum ALT for all groups rose above the values that were recorded on day zero (before DM induction) on day four (after DM induction). At the end of day 11 after different treatments, levels of serum ALT varied by slight decrease and increase according to groups in Figure 2. None of the changes in ALT after week one were statistically significant compared with ALT after alloxan injection (day 4) (all p-values>0.05). It is worth noting that the mean serum level of ALT for all diabetic groups increased, but only the mean serum level of ALT for groups IV and VI decreased ((from 65.2 to 33.7 U/L) and (33.2 U/L to 23.2 U/L), respectively). Although not statistically significant, it might suggest some hepatoprotective effects of olive leaves extract or combination extract added to insulin.

In order to determine the hepatoprotective effect of the treatments in different groups by decreasing serum levels of ALT comparative to each other, mean percentage differences on Day 11 from Day Zero were compared by conducting Kruskal-Wallis statistical test, where *p* was equal to 0.722. This indicates that there was no significant difference among the groups that received treatment.

#### Serum Alkaline Phosphatase (ALP) analysis

**Figure 3:**
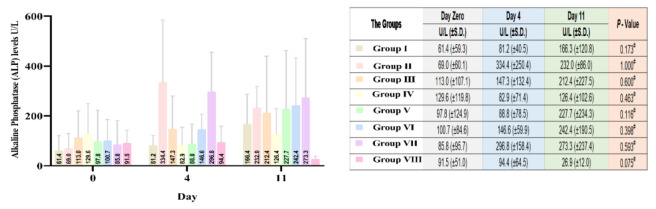
Comparative average ALP serum levels in the studied groups, Day Zero (before alloxan treatment, initial), after 4 days of the alloxan treatment (Day 4), and 1 week after different treatments (Day 11). Group I (Healthy, without treatment, n=6), Group II (Diabetic, without treatment, n=6), Group III (Diabetic, 6 IU/kg Insulin, n=7), Group IV (Diabetic, 6 IU/kg Insulin + 500 mg/kg Olive leaves extract, n=7), Group V (Diabetic,6 IU/kg Insulin + 500 mg/kg Ginger rhizome extract, n=7), Group VI (Diabetic,6 IU/kg Insulin + 500 mg/kg Olive leaves extract + 500 mg/kg Ginger rhizome extract, n=7), Group VII (Diabetic, 500 mg/kg Olive leaves extract + 500 mg/kg Ginger rhizome extract, n=6), and Group VIII (Healthy, 500 mg/kg Olive leaves extract + 500 mg/kg Ginger rhizome extract, n=6).

The mean reading of serum ALP varied between day zero and day 4 of DM induction. The variability of serum ALP readings continued until the end of day 11 after different treatments. None of the changes in ALP after week one were statistically significant (p=0.05). It is worth mentioning that the mean serum level of ALP for group VIII dramatically decreased from 94.4 to 26.9 U/L. Although not statistically significant, it might suggest some hepatoprotective effects of olive leaves and ginger extracts. To determine the hepatoprotective effect of the treatments in different groups by decreasing serum levels of ALP comparative to each other, mean percentage differences after 1 week from Day Zero were compared by conducting Kruskal-Wallis statistical test, where *p* was equal to 0.188. This indicates that there was no significant difference among the groups that received treatment (Figure 3).

### Kidney functions test

#### Serum Creatinine analysis

Creatinine levels for all groups during the experiment period were within the reference value of 0.4– 3.75 mg/dl (Burns & de Lannoy, 1966). After DM induction (Day 4), all the groups of rats experienced an elevation in serum creatinine levels. 

**Figure 4:**
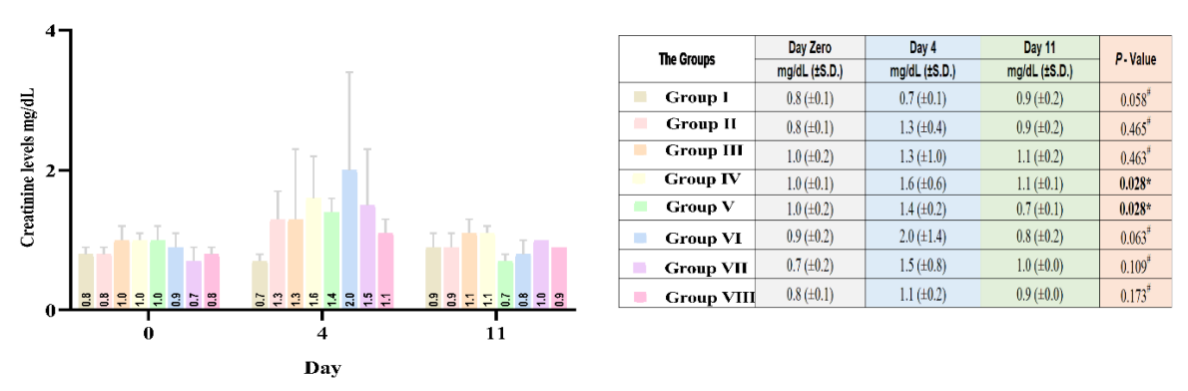
Comparative average creatinine serum levels in the studied groups, Day Zero (before alloxan treatment, initial), 4 days after alloxan treatment (Day 4), and 1 week after different treatments (Day 11). Group I (Healthy, without treatment, n=6), Group II (Diabetic, without treatment, n=6), Group III (Diabetic, 6 IU/kg Insulin, n=7), Group IV (Diabetic, 6 IU/kg Insulin + 500 mg/kg Olive leaves extract, n=7), Group V (Diabetic,6 IU/kg Insulin + 500 mg/kg Ginger rhizome extract, n=7), Group VI (Diabetic,6 IU/kg Insulin + 500 mg/kg Olive leaves extract + 500 mg/kg Ginger rhizome extract, n=7), Group VII (Diabetic, 500 mg/kg Olive leaves extract + 500 mg/kg Ginger rhizome extract, n=6), and Group VIII (Healthy, 500 mg/kg Olive leaves extract + 500 mg/kg Ginger rhizome extract, n=6).

By the end of the first week after different treatments, a decrease in serum creatinine levels occurred in most of the groups. The decrease in serum creatinine was found to be significant in two groups: group IV and group V. Rats in both groups received either olive leaves extract or ginger rhizome extract, in addition to insulin. This suggests the nephroprotective effect of the study extracts in diabetic rats (Figure 4). The combination of the two extracts could decrease serum creatinine considerably; however, no statistically significant difference was found between these two groups. In order to determine the nephroprotective effect of the treatments in different groups comparative to each other, mean percentage differences after 1 week from Day Zero were compared by conducting Kruskal-Wallis statistical test, where *p* was equal to 0.021, indicating that there was a significant difference among the groups that received treatment. Groups with less elevated serum creatinine than others were determined by One-Way ANOVA: Post Hoc Multiple Comparisons (Tukey equal variance assumption). No statistically significant difference was found between the group IV and group V than others, i.e. diabetic with insulin and olive leaves, and diabetic with insulin and ginger.

## Discussion

Considering the advantages of chemical and hormonal drugs to manage DM, drawbacks such as side effects, price, and availability are still present (Ahangarpour et al., 2017; Prajapati et al., 2024). These detriments enhanced the redirection toward the use of plants for the management of DM in many societies (Mumtaz et al., 2023). Until now, researchers have made great efforts using animal models to properly understand, the *in-vivo* features of the pharmacological activity of many herbal remedies. Therefore, today there is a long list of plants with well-known anti-diabetes activity available for either further investigation or direct use (Kifle et al., 2022).

Till now, the combination of the aqueous extracts of olive leaves and ginger rhizome had never been tested for hypoglycemic effect nor possible alleviation effect on complications associated with DM and/or its treatments. Indeed, this study can be considered impactful because it was designed to determine the effect of the extracts on two dimensions: the efficacy, represented by the ability to decrease blood glucose, and the safety, by considering potential effects on liver and kidney functions. 

Studies from the literature show that alloxan-diabetic rats have hyperglycemia due to damaging insulin-secreting cells of the pancreas (Naik et al., 2022). In this study, the 150 mg/kg single-dose intraperitoneal (I.P) of alloxan was able to build up ketone bodies in urine samples from all the rats before receiving any treatment, comparing the beige strips with the reference color chart provided in the kit. Hence, according to Chowdhury and Chakraborty, this most probably demonstrates diabetic ketoacidosis which usually appears in more than 90% of T1DM cases when they do not take exogenous insulin (Chowdhury and Chakraborty, 2022). This finding was consistent with findings from other studies (Ben et al., 2019; Tanko et al., 2014), and thus supports that this dose of alloxan had induced T1DM in the study rats, which can never be treated without insulin injection. This ketoacidosis danger may be the response behind a high death in rats after alloxan injection in this study. 

Based on the study of Abu-zaiton and Abu-Albasal, water decoction of olive leaves at a dose (133.3 mg/ kg) orally once a day for 14 days could significantly reduce blood glucose in normal and alloxan-induced diabetic Wistar rats (Abu-zaiton and Abu-Albasal, 2012b). Zoair reported that an aqueous extract of olive leaves at a dose of 7.5 mg/kg orally twice daily for 30 days had a hypoglycemic effect in alloxan–diabetic Albino rats (Zoair, 2014). Other studies tried to explain the acute hypoglycemic effect of olive leaves extract as probably because olive leaf-derived polyphenols, specifically oleuropein were associated with improved glucose metabolism via inhibition of pancreatic amylase activity, thus inhibiting starch digestion, inhibiting glucose uptake, stimulating hepatic glycogen synthesis, and as a result, reducing hyperglycemia (Alhujaily et al., 2022; Golovinskaia and Wang, 2023). Elshater and colleagues reported that treatment of alloxan-induced diabetic Wistar rats with 4 ml/kg ginger juice orally for 6 weeks could significantly reduce plasma glucose levels. Although that reduction in plasma glucose was not enough to reach normal levels, it was still lower when compared with the control group (Elshater et al., 2018). The possible mechanism of action of ginger as a glycemic regulator might include inhibition of α-amylase and α-glucosidase enzymes in carbohydrate digestion, and/or increase of insulin release (Prabhakar et al., 2014; Bi et al., 2017). 

In the present study, the healthy group treated with this combination exhibited a significantly decreased level of FBG compared to the control group. Moreover, the diabetic group treated with this combination and insulin exhibited a significantly decreased level of FBG compared to diabetic groups without treatment and the diabetic group treated with this combination alone. The reduction of FBG in the four diabetic groups that were treated (insulin only, insulin and olive leave aqueous extract, insulin and ginger aqueous extract, and insulin and the combination) was not enough to reach FBG levels in the control group, still the FBG.

Although the combination of aqueous extracts of olive leaves and ginger rhizome without insulin could not fight the high FBG in diabetic rats, it showed a powerful effect in reducing FBG in healthy rats. This combination without insulin had not any effect on the diabetic rats could be due to losing the key of its mechanism, to clarify the whole β-cells were killed by alloxan as other studies had mentioned before (Abu-zaiton and Abu-Albasal, 2012b). Hence, it could not stimulate insulin release.

Alloxan enters the β-cells by using glucose transporter (2 GLUT2) due to its hydrophilic nature. Likewise, it can enter hepatocytes and kidney tubular cells which express GLUT2 transporters (Feda, 2022; Radenković et al., 2016), this leads to elevated ALT, ALP, and creatinine in diabetic rats due to the effect of reactive oxygen species (ROS) decreasing in both enzymatic and non-enzymatic antioxidant activities (El Sadek et al., 2016); this explains why almost all diabetic rats groups in this study, faced the elevation of liver and kidney toxicity markers on day 4 after alloxan injections. *Mouse and colleague* reported that treatment of alloxan-induced diabetic rats with olive leaves extract for 6 weeks, significantly reduced plasma ALT levels (Mousa et al., 2014); additionally, *El Sadek* and colleagues mentioned that treatment of streptozotocin-induced diabetic rats with the basal diet with 15% of ginger for 30 days, showed significantly reduced plasma ALT level (El Sadek et al., 2016). 

The current results indicated that one week after different treatments, the olive leave extract was able to keep ALP level under the reference value≤159 U/L (Wi, 2008) only in the diabetic group which received (6 IU/kg insulin + 500 mg/kg olive leaves extract) and the healthy group which received (500 mg/kg olive leaves extract + 500 mg/kg ginger rhizome extract) experienced a severe drop in ALP levels compared to the control group. 

Afify et al. reported that treatment of streptozotocin-induced diabetic rats with olive leaves extract orally for 10 weeks, showed a significant reduction in creatinine levels post-therapy (Afify et al., 2018). Likewise, *El-Kott* and his colleague mentioned that treatment of alloxan-induced diabetic rats with ginger rhizome extract at a dose of 400 mg/kg orally once daily for 4 weeks, showed a significant reduction in creatinine levels on the last day of treatment (El-Kott et al., 2010).

Here, the results agree with findings from former studies; it was found that aqueous extracts of olive leaves and ginger rhizome administered alone with insulin significantly reduced the serum creatinine level. 

These two plants contain a high phenol content and have strong antioxidants which increase the total serum antioxidant capacity, thus increasing the ability to scavenge free radicals and finally, prevent liver and kidney damage (El Sadek et al., 2016; El-Kott et al., 2010; El-Beltagi, 2018; Kazemian et al., 2015; Mousa et al., 2014).

Finally, when DM is addressed in research areas, the mortality rate becomes the focus of attention as many studies aim to find a natural drug that can improve the quality of life of diabetic patients; thus, extending their life, when it is added to synthetic drugs.

In this current study, it was found that the diabetic group that received olive leaves extract, ginger rhizome extract and 6 IU/kg insulin had notably better survival rates than other diabetic groups after 14 days of treatment. 

 Here, although insulin with the aqueous extracts of olive leaves, ginger rhizome, and their combination was able to reduce the FBG in diabetic type 1 Wistar rats after one week of treatment, of there was not any statistically significant differences between each other or between them and the diabetic group who received insulin alone

This combination with insulin had a powerful effect in improving the mortality rate in diabetic rats over other groups in this research. As could be expected, and since alloxan had most probably induced T1DM, this combination alone without insulin was not able to reduce high FBG in diabetic rats and improve the mortality rate. However, this combination exhibited a potent decreasing effect on the FBG in the healthy group. The results showed that insulin with the aqueous extracts of olive leaves, and a combination of extracts of olive leaves and ginger rhizomes were able to reduce the serum levels of ALT in alloxan-induced diabetic rats. Moreover, the aqueous extracts of olive leaves and a combination of extracts of olive leaves and ginger rhizomes were able to keep the serum levels of ALP within the normal range for the diabetic group and healthy group, respectively. Additionally, the results demonstrated that insulin with olive leaves extract and ginger rhizome extract each alone significantly reduced creatinine levels in alloxan-induced diabetic type 1 Wistar rats compared to the combinations with/without insulin one week of treatment. However, the short life (18 days) of this study remains its main setback.

**Table 1:** The experiment groups, and the treatments that were used

Groups Numbers	Status	Classification	Treatments	Total number of rats	Number of Males	Number of Females
**Group I**	Healthy	Negative Control group	Without Treatment	6	3	3
**Group II**	Diabetic	Positive Control group	Without Treatment	6	3	3
**Group III**	Diabetic	Test group	6 IU/kg Insulin	7	3	4
**Group IV**	Diabetic	Test group	6 IU/kg Insulin + 500 mg/kg Olive leaves extract	7	3	4
**Group V**	Diabetic	Test group	6 IU/kg Insulin + 500 mg/kg Ginger rhizome extract	7	3	4
**Group VI**	Diabetic	Test group	6 IU/kg Insulin + 500 mg/kg Olive leaves extract + 500 mg/kg Ginger rhizome extract	7	3	4
**Group VII**	Diabetic	Test group	500 mg/kg Olive leaves extract + 500 mg/kg Ginger rhizome extract	6	3	3
**Group VIII**	Healthy	Test group	500 mg/kg Olive leaves extract + 500 mg/kg Ginger rhizome extract	6	3	3

**Table 2:** Comparative mean FBG levels (mg/dl) in the studied groups, Day Zero (before alloxan treatment, initial), 4 days after alloxan treatment (day 4), and 1 week after different treatments (day 11).

The Groups	Glucose (Day Zero) mg/dL mean (±S.D.)	Glucose (Day 4) mg/dL mean (±S.D.)	Glucose (Day 11) mean (±S.D.)	p- Value
**Group I**	62.5 mg/dl (±6.5)	65.7 mg/dl (±3.7)	70.8 mg/dl (±6.5)	0.116^#^
**Group II**	65.7 mg/dl (±3.7)	330.2 mg/dl (±229.7)	249.5 mg/dl (±100.1)	0.285^#^
**Group III**	66.3 mg/dl (±3.7)	282.0 mg/dl (±148.0)	132.3 mg/dl (±103.7)	0.028*
**Group IV**	66.3 mg/dl (±3.7)	385.7 mg/dl (±207.7)	122.2 mg/dl (±78.8)	0.028*
**Group V**	66.3 mg/dl (±3.7)	204.8 mg/dl (±107.4)	109.0 mg/dl (±36.1)	0.027*
**Group VI**	66.3 mg/dl (±3.7)	302.9 mg/dl (±219.4)	144.4 mg/dl (±102.2)	0.028*
**Group VII**	65.7 mg/dl (±3.7)	292.3 mg/dl (±214.9)	495.0 mg/dl (±105.6)	0.109^#^
**Group VIII**	62.5 mg/dl (±6.5)	85.2 mg/dl (±8.5)	66.3 mg/dl (±9.5)	0.027*

*Statistically significant difference (p≤0.05), ‘’Wilcoxon Signed Ranks Test (nonparametric)’’. ^#^Statistically insignificant difference (p≥0.05), ‘’Wilcoxon Signed Ranks Test (nonparametric)’
